# Based on C-CHEWS study on the application value of comprehensive perioperative nursing based on scoring in critically ill children with pediatric cardiovascular surgery

**DOI:** 10.3389/fped.2025.1605731

**Published:** 2025-09-02

**Authors:** Feifei Li, Aifang Mao, Xiaomei Niu, Qingqing Zhou

**Affiliations:** Department of Pediatric Cardiovascular Surgery, Anhui Hospital, Children’s Hospital Affiliated to Fudan University, Anhui Provincial Children’s Hospital, Hefei, China

**Keywords:** C-CHEWS score, comprehensive nursing, cardiovascular surgery, critically ill children, early identification

## Abstract

**Objective:**

To assess the association of comprehensive perioperative nursing, including early identification based on the Children's Cardiovascular Early Warning Scale (C-CHEWS score), with outcomes in critically ill children undergoing pediatric cardiovascular surgery in an observational setting.

**Methods:**

This study analyzed data from 120 children with congenital heart disease admitted to the Department of Pediatric Cardiovascular Surgery between March 2020 and March 2022. Patients were categorized based on the type of nursing care received: routine care or nursing care incorporating C-CHEWS scoring. The length of hospital stay, pediatric intensive care unit (ICU) monitoring time, disease control status (death, deterioration, and stability), quality of life, incidence of adverse events, and family members’ satisfaction were recorded and analyzed.

**Results:**

The observed ICU monitoring time and hospitalization duration were significantly shorter in the comprehensive care group (*P* < 0.05). Disease control rates were higher in the comprehensive care group (76.67%) compared to the routine care group (30.0%) (*P* < 0.05). Quality of life scores at discharge were improved in the comprehensive care group (*P* < 0.05). The incidence of adverse events was lower in the comprehensive care group (10.00%) compared to the routine care group (46.67%) (*P* < 0.05). Family satisfaction with nursing care was higher in the comprehensive care group (96.67%) compared to the routine care group (70.0%) (*P* < 0.05).

**Conclusion:**

The comprehensive perioperative nursing approach, which included the C-CHEWS score, was associated with earlier identification of clinical deterioration, reduced adverse events, and improved family satisfaction in critically ill children undergoing pediatric cardiovascular surgery. These findings support the potential for broader adoption of this nursing model in clinical practice.

## Introduction

Children with congenital heart disease (CHD) often face critical challenges due to their complex cardiovascular conditions, particularly during the perioperative period (1). Perioperative nursing plays a vital role in ensuring early detection of clinical deterioration, effective management of complications, and optimization of recovery. However, due to the young age and limited ability of these patients to communicate symptoms, high-quality perioperative care is crucial to improve survival rates and quality of life outcomes in this vulnerable population (2–4). A common congenital deformity, congenital heart disease (CHD) makes about 28% of all congenital malformations. Its anatomical defects in the heart and larger blood arteries found during embryonic development or from the failure of normal postnatal closure of particular channels define it (1, 2). Though the exact etiology of CHD is yet unknown, its possible effects are rather serious. Children with CHD are quite likely to have consequences including heart failure and often show symptoms including dyspnea, edema, too much sweating and tiredness (3–5). Urgent medical care is needed when the state of a child with CHD deteriorates. Once their health gets worse, the success rate of treatment in critically ill children stays low even with developments in medical technology that enable constant monitoring (6–8).

Comprehensive nursing care, which integrates dynamic monitoring tools such as the C-CHEWS scoring system, has gained attention for its ability to identify early warning signs in critically ill children (9). Unlike traditional nursing methods, this approach incorporates specific cardiovascular indicators tailored to CHD patients, enabling precise interventions. Despite its potential, the clinical application of comprehensive care using C-CHEWS remains underexplored in many regions, particularly in resource-constrained settings (10). There is limited evidence assessing its effectiveness in reducing complications and improving outcomes, highlighting a gap in the literature.

Improving prognosis in critically ill children depends on a quick intervention, hence a useful and quick assessment tool is absolutely vital. Such a tool can help to standardize treatment, lower variation in clinical judgment depending on expertise and encourage efficient communication among healthcare providers at several stages of treatment (11, 12). Though important, widely used severity assessment measures include the Pediatric Intensive Care Score (PCIS) and the Pediatric Mortality Risk Score (PRISM) are typically complicated and call for particular laboratory tests and equipment. Their limited applicability is especially true in rural or resource- constrained environments (13, 14).

By Monaghan in 2005, the Pediatric Early Warning Score (PEWS) presents a simpler, more easily available substitute. With three main physiological criteria, consciousness, cardiovascular function and respiratory status, each evaluated on a scale from 0 to 3 PEWS evaluates children. In Western healthcare systems, where it has been demonstrated to help early identification of clinical deterioration and lower pediatric mortality, this instrument has become rather common. PEWS has restrictions for children with congenital cardiac problems even if it is rather successful. PEWS does not fairly portray the clinical symptoms of children with CHD, according a study by McLellan et al. from Boston Children's Hospital (12, 15, 16).

This study aims to evaluate the effectiveness of a comprehensive perioperative nursing model based on the C-CHEWS scoring system in critically ill children undergoing pediatric cardiovascular surgery. By comparing this model with conventional nursing care, the study seeks to provide robust evidence supporting its role in improving clinical outcomes, reducing adverse events, and enhancing patient-family satisfaction.

## Materials and methods

### Study design and setting

This cross-sectional observational study was conducted between March 2020 and March 2022 at the Department of Pediatric Cardiovascular Surgery in our hospital (Approval No. 1439/FU dated January 03, 2020). We included 120 critically ill children diagnosed with congenital heart disease who underwent pediatric cardiovascular surgery. Patients were categorized into two groups based on the type of care received: routine care and comprehensive care, which incorporated the C-CHEWS scoring system. The routine care group received standard perioperative care, while the comprehensive care group received additional early detection and intervention based on C-CHEWS scoring. The groups were not formed through random allocation or propensity score matching (PSM). Instead, the allocation was based on hospital records, distinguishing patients who received routine perioperative care from those who received comprehensive care incorporating the C-CHEWS scoring system. Patients were classified into two groups based on the nursing care they received during hospitalization: the routine care group (60 children: 34 males and 26 females, mean age 4.85 ± 1.29 years) and the comprehensive care group (60 children: 31 males and 29 females, mean age 4.71 ± 1.63 years).

### Inclusion and exclusion criteria

Children were eligible for the study if they were diagnosed with CHD via ultrasound, classified as critically ill post-surgery, and aged between 3 months and 10 years. Only children without cognitive impairments and who were capable of complying with nursing interventions were included. Exclusion criteria comprised children diagnosed with mental illness, newborns (within 28 days), those who passed away before hospitalization, those transferred to another hospital or who discontinued treatment, and those enrolled in other clinical studies during the same period.

### Nursing approaches

Routine Care Group: Children who received standard perioperative care, which included general nursing interventions such as regular monitoring of vital signs, maintaining airway patency through suctioning, and performing nebulization as needed. Routine care also included monitoring consciousness and respiratory symptoms, using a ventilator when required, and following aseptic protocols for equipment use. Antibiotics and nutritional support were observed as per medical prescriptions to prevent secondary infections.

Nursing Care Group: In addition to routine care, children in this group were managed using a comprehensive nursing protocol that incorporated the C-CHEWS score for early detection and intervention. Key components of the comprehensive care included:

Early Warning Assessment: Nurses regularly assessed and recorded vital signs such as consciousness, heart rate, oxygen saturation, and respiration. Based on C-CHEWS scores, interventions were categorized into graded actions.

### Graded interventions

1. C-CHEWS Score < 3: No special intervention, with only dynamic monitoring of vital signs.
2. C-CHEWS Score = 3: Senior nursing staff was alerted for daily or dynamic assessment over three days.
3. C-CHEWS Score = 4: Doctors were notified, and monitoring was increased with assessments every 8 hours.
4. C-CHEWS Score ≥ 5: Intensive care was initiated with 24-hour monitoring, timely suctioning, and oxygen administration. Additional support such as medication and continuous assessment was provided as per the clinical status.

### Comprehensive nursing involvements

Vital Monitoring: Close monitoring of pupil response, consciousness levels, and intracranial pressure. Immediate notification to the physician if abnormal vital signs or increased intracranial pressure were detected.

Psychological Support: Nurses provided psychological counseling to both the child and their family, explaining the disease and treatment process to reduce anxiety and improve cooperation.

Environmental Management: Ensured a clean environment, proper temperature control, and rescue equipment availability.

Temperature and Diet Care: Body temperature was monitored every 4 hours. Physical cooling or medications were observed for high fever, while nutritional support was given according to the child's condition, progressing from fluids to a low-salt, low-oil semi-liquid diet.

Other Monitoring: Attention was paid to bowel movements, stool characteristics, and any abnormal conditions such as constipation.

### Observation indicators

The following parameters were measured to evaluate the outcomes:

Length of Hospital Stay and ICU Monitoring Time: The total hospitalization duration and time spent in the Pediatric Intensive Care Unit (ICU) were recorded for both groups.

Disease Control: Children were categorized based on whether their condition worsened, stabilized, or resulted in death during hospitalization. Disease control was assessed by changes in C-CHEWS scores. The effective disease control rate was calculated as:

Effective disease control rate = (Number of cases with stable disease/Total number of cases) × 100.

Quality of Life: Quality of life was measured using a life coefficient score, which evaluated factors such as energy level, pain, emotional response, sleep quality, social isolation, and physical activity. Lower scores indicated better outcomes. Assessments were performed at admission and discharge.

Quality of life was assessed using validated life coefficient score, which measured six domains: energy level, pain, emotional response, sleep quality, social isolation, and physical activity. Each domain was scored on a scale ranging from 0 to 10, where lower scores indicated better outcomes. The specific scoring ranges were as follows:

Energy Level: 0 (high energy) to 10 (extremely fatigued).

Pain: 0 (no pain) to 10 (severe pain).

Emotional Response: 0 (calm and stable) to 10 (extremely distressed).

Sleep Quality: 0 (excellent sleep) to 10 (severely disturbed sleep).

Social Isolation: 0 (not isolated) to 10 (highly isolated).

Physical Activity: 0 (fully active) to 10 (severely limited activity).

Adverse Events: Incidents such as arrhythmias, unplanned extubations, falls, and the loss of monitoring equipment were recorded. The total incidence of adverse events was calculated as:

Total incidence of adverse events = (Number of adverse events/Total number of cases) × 100.

Family Satisfaction with Nursing Care: A 10-item nursing satisfaction questionnaire was filled from the family members of the children before discharge. Satisfaction levels were categorized based on a 100-point scale, with higher scores indicating greater satisfaction.

## Statistical analysis

All statistical analyses were performed using SPSS 22.0 software. Continuous variables were expressed as mean ± standard deviation (SD) and compared between groups using the independent-sample t-test. Categorical data were presented as frequencies and percentages, with differences between groups assessed using the chi-square test. To enhance the interpretation of the results, 95% confidence intervals (CIs) were reported alongside *p*-values. Statistical significance was defined as *p* < 0.05.

## Results

### Patient demographics and baseline characteristics

Baseline characteristics of the patients were analyzed, including age, gender, preoperative diagnosis, and severity of congenital heart disease. The mean age was 4.78 ± 1.46 years, with an approximately equal distribution of males and females across both groups. Baseline characteristics were comparable between the comprehensive care and routine care groups (*P* > 0.05).

### Length of hospital stay and ICU care time

Compared to the Routine Care Group (20.5 ± 3.2 days and 9.5 ± 1.8 days, respectively), Nursing Care Group had notably shorter hospitalization periods (13.2 ± 2.1 days) and ICU monitoring times (6.8 ± 1.4 days). The *P*-values of 0.002 for hospitalization time and 0.023 for ICU monitoring time point to statistically significant variations across the groups. These results implied that the whole nursing care strategy successfully shortens the length of hospital stays and ICU monitoring in children under pediatric cardiovascular surgery who are severely unwell (Table 1).

**Table 1:** Comparison of ICU monitoring time and hospitalization duration between the comprehensive care group and routine care group.

Group	Hospitalization time (Mean ± SD)	ICU monitoring time (Mean ± SD)	*P*-value
Routine care group	20.5 ± 3.2	9.5 ± 1.8	0.002
Nursing care group	13.2 ± 2.1	6.8 ± 1.4	0.023

Data are presented as mean ± standard deviation. Significant differences are indicated by *p* < 0.05.

### Disease control comparison

**Figure 1:**
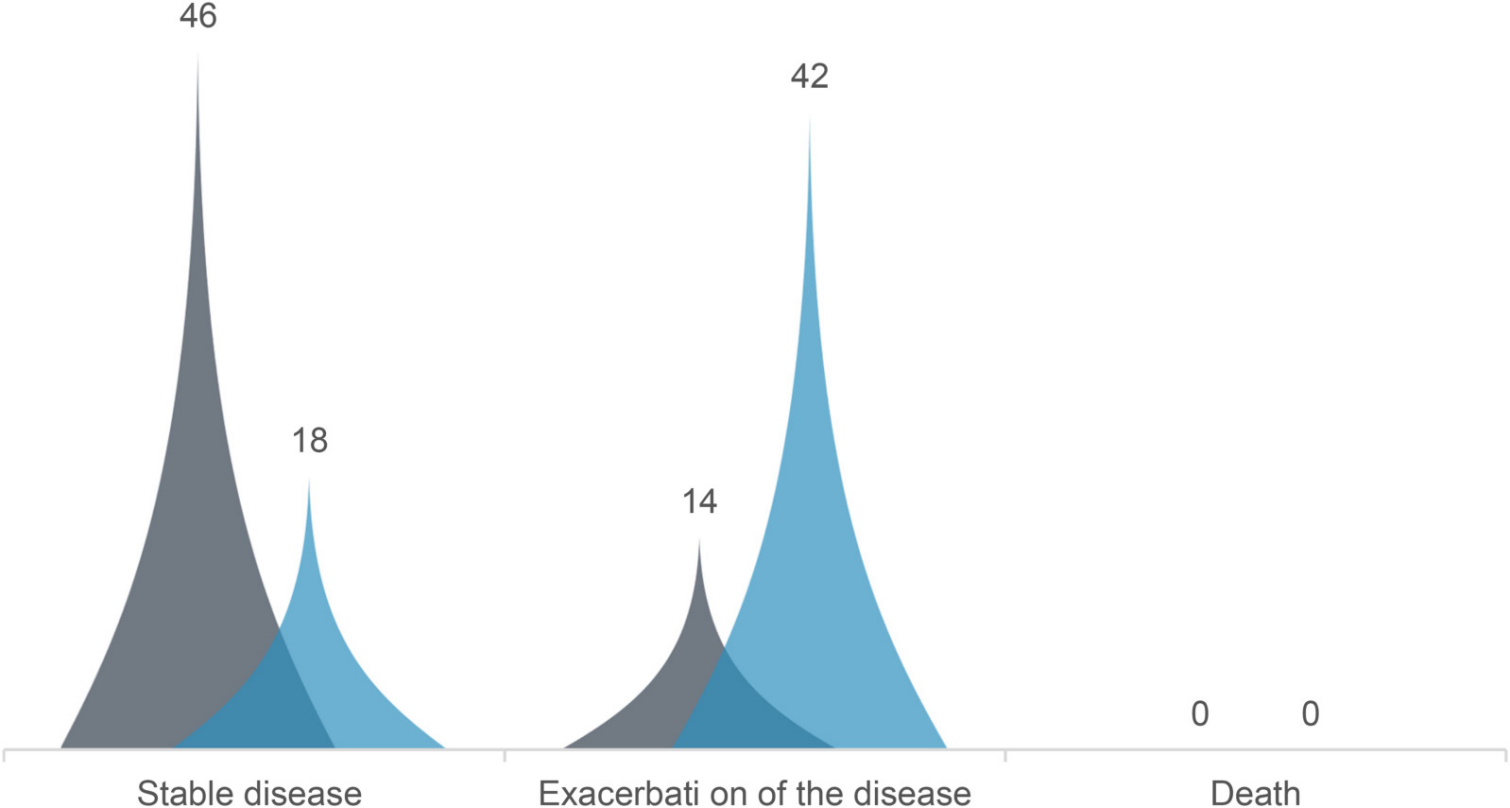
Comparison of disease control between the comprehensive care group and the routine care group. The comprehensive care group exhibited significantly higher disease control rates (76.67% vs. 30%) with a *P*-value of < 0.05. Blue: comprehensive care group; Gray: routine care group.

In the observed standard care group, 18 cases had stable disease, 42 cases had aggravated disease, and no deaths were reported. In the observed comprehensive care group, 46 cases had stable disease, 14 had aggravated disease, and no deaths were reported, with a significantly higher effective disease control rate in the comprehensive care group (76.67%) compared to the standard care group (30.0%) (*P* < 0.05) (Figure 1).

### Quality of life scores before and after the intervention

Before the intervention, there was no significant difference observation in the scores of various life quality between the two groups (*P* > 0.05), but after the intervention, the scores of various life coefficients in the comprehensive care group were significantly lower than those to conventional group, and the difference between the groups was statistically significant (*P* < 0.05) (Table 2).

**Table 2:** Life coefficient scores before and after intervention ($\bar{x}\pm s$, points) coefficient items.

Group	*N*	Energy level		pain		Emotional Response		Sleep	
		Before intervention	After intervention	Before intervention	After intervention	Before intervention	After intervention	Before intervention	After intervention
Routine care group	60	8.51 ± 2.15	6.83 ± 2.04^a^	5.56 ± 0.28	4.01 ± 0.23 ^a^	7.81 ± 1.29	5.58 ± 1.04^a^	10.23 ± 2.56	8.34 ± 1.03^a^
Nursing care group	60	8.64 ± 2.07	1.07 ± 0.09^b^	5.48 ± 0.37	0.93 ± 0.12^b^	7.69 ± 1.32	0.48 ± 0.09^b^	10.21 ± 2.48	4.6 ± 0.7^b^
*t*		0.337	21.850	1.335	91.964	0.504	37.844	0.043	59.124
*P*		>0.05	<0.05	>0.05	<0.05	>0.05	<0.05	>0.05	<0.05

Comparison of observed standard care group before and after treatment.

^a^ *P* < 0.05; comparison of observed nursing care group before and after treatment.

^b^ *P* < 0.05.

**Table:** 

Group	*N*	Social isolation		Physical activity	
		Before intervention	After intervention	Before intervention	After intervention
Routine care group	60	0.56 ± 0.07	0.55 ± 0.02	7.49 ± 1.34	5.62 ± 2.23^a^
Nursing care group	60	0.54 ± 0.08	0.51 ± 0.03^b^	7.88 ± 1.27	0.72 ± 0.18^b^
*T*		1.457	8.593	1.636	16.965
*P*		>0.05	<0.05	>0.05	<0.05

Comparison of the observed standard care group before and after treatment.

^a^ *P* < 0.05; comparison of observed comprehensive care group before and after treatment.

^b^ *P* < 0.05.

### Comparison of the incidence of adverse events between groups

The incidence of adverse events of observed comprehensive care group was 10.00%, which was lower than that in the observed standard care group (46.67%), and the difference between the two groups was statistically significant (*P* < 0.05) (Table 3).

**Table 3:** Comparison of adverse event incidence among groups [*n* (%)].

Group	*N*	Arrhythmias	Low cardiac output syndrome	Unplanned extubation	Falling/falling out of bed	Total incidence (%)	*p*-value
Routine care group	60	11 (1 8.33)	7 (11.67)	6 (10.00)	4 (6.67)	28 (46.67)	<0.05
Nursing care group	60	3 (5.00)	1 (1.67)	0 (0.00)	2 (3.33)	6 (10.00)	

### Comparison of family members’ satisfaction with nursing care

The satisfaction of family members of children with nursing care in t observed standard care group was 70.0% and observed comprehensive care group was 96.67%. The difference in satisfaction of family members of children with nursing care between the two groups was statistically significant (*P* < 0.05) (Table 4).

**Table 4:** Comparison of family members ‘ satisfaction with nursing care.

Group	*N*	Very satisfied	Satisfied	Basically satisfied	Dissatisfied	Satisfaction	*χ2*	*p*-value
Routine care group	60	15 (25.00)	17 (28.33)	10 (16.67)	18 (30.00)	42 (70.00)	15.360	<0.05
Nursing care group	60	26 (43.33)	2 0 (33.33)	12 (20.00)	2 (3.33)	58 (96.67)		

## Discussion

This study adopted an observational research design to systematically compare comprehensive care, which incorporated the C-CHEWS scoring system, with conventional care methods. By analyzing key indicators such as ICU monitoring time, hospitalization duration, disease control rates, adverse event incidence, quality of life, and family satisfaction, the study provided robust evidence of the benefits of comprehensive care. The findings highlighted the potential of integrating the C-CHEWS score into perioperative nursing protocols to improve clinical outcomes in critically ill children undergoing pediatric cardiovascular surgery. These results offered valuable insights for optimizing clinical practice and underline the importance of broader implementation of this nursing model in similar settings.

With the improvement of cardiac surgical techniques and the improvement of perioperative monitoring, the age of patients undergoing surgery has gradually become younger. As the patients are young, their consciousness and behavior are poor, and their treatment cooperation is poor, coupled with the complex conditions of the children and the heavy nursing tasks, higher requirements are placed on the quality of nursing services. Most pediatric patients need timely treatment after admission, supplemented by high-quality nursing services, in order to save their lives to the greatest extent possible and reduce the occurrence of related complications and sequelae. Studies have shown that 26% to 43% of pediatric deaths are avoidable (17–19).

Nursing and treatment complement each other and are indispensable. To some extent, the role of nursing is far greater than that of treatment, especially for critically ill children (20). The vast majority of critically ill children lack the ability to take care of themselves, which places greater demands on clinical care (21). Conventional monitoring measures cannot meet the actual needs or nursing expectations of parents (22). Clinical studies have reported that there are early warning signs that can be monitored before the condition of most critically ill children changes dramatically. In view of this, it is of great significance to seek a more effective and accurate disease scoring tool.

Since children with critical congenital heart disease are relatively young and their condition changes rapidly, they need a sensitive, accurate, and applicable clinical scoring tool. Currently, there are many disease scoring tools, but these scoring tools are widely used and are not suitable for children with congenital heart disease. In addition, some indicators are difficult to collect, lack dynamic evaluation, and cannot obtain continuous scoring results. Therefore, the clinical guidance significance for early warning of congenital heart disease is not very strong (23). Children with congenital heart disease urgently need a set of sensitive, accurate, and applicable scoring tools. The PEWS score combines the characteristics of children's diseases and evaluates vital signs such as consciousness, providing nurses with an objective and reasonable scoring tool to determine the condition of the child. On the other hand, the C-CHEWS score is a special evaluation tool based on the PEWS score to judge the changes in the condition of children with congenital heart disease, providing a standardized evaluation and treatment nursing process for the clinic. At present, the application of this evaluation tool in China is still in its infancy, and there are few reports on its clinical application value. This study adopted a perioperative comprehensive nursing method based on the C-CHEWS score and applied it to the clinical care of critically ill children in pediatric cardiovascular surgery.

C- CHEWS score in observed comprehensive care group was shorter than that in observed standard care group and the effective control rate of the disease in observed comprehensive care group was higher than that in observed standard care group. It is suggested that the application of C-CHEWS score can quickly and accurately evaluate the condition of children with congenital heart disease to obtain the best treatment window, which has a positive effect on shortening the treatment time and hospitalization time and improving the condition of children (23). The incidence of related adverse events in observed comprehensive care group was low, indicating that C-CHEWS score management can reduce the incidence of adverse events and reduce secondary harm to children. The possible reason is that C-CHEWS score can better predict the risk of disease progression in children and guide clinical targeted measures to avoid the occurrence of adverse events (7). After the children are admitted to the hospital, C-CHEWS dynamic assessment is continuously carried out to monitor the changes in the children's condition in real time, inform the doctor in time, and deal with it in time. Children can receive timely and effective treatment, thereby reducing the number of adverse events, improving the prognosis of children and increasing the satisfaction of children's families with care (12).

The reduction in complications such as arrhythmias and low cardiac output syndrome in the comprehensive care group is likely due to the early detection and timely interventions enabled by the C-CHEWS scoring system. Comprehensive care emphasizes continuous monitoring, graded responses, and targeted management, which prevent complications from escalating. While surgical techniques are crucial, these complications are more often linked to postoperative management, where comprehensive care plays a pivotal role in stabilizing patients and improving outcomes.

The scores of the life coefficients in observed comprehensive care group were significantly lower than observed standard care group. Family members in observed comprehensive care group were more satisfied with the nursing. The specific reason is that the application of C-CHEWS score management can timely and accurately grasp the changes in the child's condition, and cooperate with scientific intervention measures to improve the treatment effect (8, 11). Secondly, it can significantly improve the utilization rate of medical resources (14). Pediatric cardiovascular surgery has high technical requirements and is prone to shortages of manpower and material resources. The application of C-CHEWS score can reduce resource waste to a certain extent, greatly improve the hospital's ability to assess children's diseases and help improve the work ability and efficiency of nurses. Nurses can judge and identify the child's condition early based on the C-CHEWS score, give parents professional guidance, help improve the overall work efficiency of the child during hospitalization, and provide higher quality nursing services for the child and his family. Finally, it can improve the cooperation of the child and his family with the treatment and the satisfaction of nursing. The application of C-CHEWS score can help medical staff accurately assess the child's condition and carry out targeted treatment, which is conducive to protecting their physical and mental health, and has been recognized by many children and their families. Related studies have also shown that the C-CHEWS score can help assess the condition of hospitalized children with congenital heart disease and identify them early, make timely judgments on treatment, improve family members ‘ satisfaction with care, and establish a harmonious nurse-patient relationship (14, 23).

This study leveraged the C-CHEWS scoring system, a specific and tailored assessment tool for children with congenital heart disease, to identify and grade clinical interventions. Unlike general pediatric early warning systems, the C-CHEWS score is designed to better reflect the unique cardiovascular symptoms associated with congenital heart disease. Its application enabled early detection of clinical deterioration and facilitated graded interventions based on the severity of the condition. This targeted approach contributed to improved patient outcomes, reduced adverse event incidence, and enhanced the overall quality of care, underscoring the significance of utilizing specialized assessment tools in pediatric cardiovascular nursing practices.

The findings of this study support the potential integration of the C-CHEWS score into routine perioperative care for critically ill pediatric patients undergoing cardiovascular surgery. The early identification of clinical deterioration enabled by this model could lead to more timely interventions, reduce ICU stays, and improve patient outcomes. Future studies should consider controlling for confounding variables through multivariate analysis to further strengthen the robustness of the results.

### Limitations

While this study provides valuable insights into the application of the C-CHEWS score in pediatric cardiovascular care, it is important to acknowledge several limitations inherent in the observational design. The lack of randomization and propensity score matching may have introduced selection bias, potentially influencing the results. Additionally, the study was conducted at a single center with a relatively small sample size (*n* = 120), which may limit the generalizability and statistical power of the findings. Therefore, caution should be exercised when applying these results to broader populations. To enhance the external validity of these findings, future studies with randomized controlled designs or propensity score matching would be beneficial. Furthermore, multicenter studies with larger and more diverse patient populations are needed to validate these results and confirm the applicability of the comprehensive perioperative nursing model, incorporating the C-CHEWS scoring system, across different healthcare settings.

## Conclusion

This study demonstrates that the comprehensive perioperative nursing model, incorporating the C-CHEWS scoring system, significantly improves clinical outcomes, reduces adverse events, and enhances family satisfaction. These findings suggest that the C-CHEWS score could be incorporated into routine pediatric cardiovascular care to improve early detection and patient management. These findings provide robust evidence to support the clinical promotion and broader adoption of this care model in pediatric cardiovascular nursing. We observed that the use of the C-CHEWS score in comprehensive perioperative care for critically ill children undergoing pediatric cardiovascular surgery was associated with earlier detection of clinical deterioration and timely intervention. This approach was linked to more precise treatment and care, a lower incidence of adverse events, and higher family satisfaction with nursing care. These findings suggest the potential value of incorporating this nursing model in clinical settings.

## Data Availability

The original contributions presented in the study are included in the article/Supplementary Material, further inquiries can be directed to the corresponding author.
